# Complete Genome Sequence of *Magnetospirillum* sp. ME-1, a Novel Magnetotactic Bacterium Isolated from East Lake, Wuhan, China

**DOI:** 10.1128/genomeA.00485-17

**Published:** 2017-08-24

**Authors:** Linfeng Ke, Pengming Liu, Shan Liu, Meiying Gao

**Affiliations:** a Wuhan Institute of Virology, Chinese Academy of Sciences, Wuhan, People’s Republic of China; b University of Chinese Academy of Sciences, Beijing, People’s Republic of China

## Abstract

A novel spiral magnetotactic bacterium, *Magnetospirillum* sp. ME-1, was isolated from East Lake in China. Here we report the complete genome of ME-1, which contains a 4,551,873-bp circular chromosome and a 5,222-bp circular plasmid. The magnetosome biogenesis-specific genes are located in a 97,664-bp magnetosome genomic island.

## GENOME ANNOUNCEMENT

Magnetotactic bacteria (MTB) are a group of widely distributed motile prokaryotes that produce magnetosomes, which consist of magnetic nanoparticles (1). Magnetosomes are attractive materials for applications in nanotechnology, biomedicine, and other fields (2). The formation of magnetosomes is strictly controlled by the magnetosome island (MAI) (3). To date, complete MTB genome sequences have been reported for just seven strains: *Magnetospirillum magneticum* AMB-1, *Magnetospirillum gryphiswaldense* MSR-1, *Magnetospira* sp. QH-2, *Magnetospirillum* sp. XM-1, *Magnetococcus marinus* MC-1, “*Candidatus* Magnetococcus massalia” MO-1, and *Desulfovibrio magneticus* RS-1 (4–10). Furthermore, the molecular mechanisms of magnetosome formation are far from fully understood. To further understand and advance the application of MTB, we present here the complete genome sequence of *Magnetospirillum* sp. ME-1.

Strain ME-1, a novel spiral MTB strain, was isolated from East Lake in Wuhan, People’s Republic of China. The genome of ME-1 was sequenced using the Illumina HiSeq 2500 platform by the paired-end method (2 × 150 bp) with a coverage of 400×. A complete genome was obtained by *de novo* assembly (11) combined with genome walking with a total length of 4,551,873 bp and a G+C content of 65.63%. In addition, a 5,222-bp circular plasmid with a 60.67% G+C content was found.

Phylogenetic 16S rRNA analysis revealed that ME-1 is closely related to *M. magneticum* AMB-1. Furthermore, genome annotation was performed using the Prokaryotic Genome Automatic Annotation Pipeline (12). The ME-1 genome contains 4,086 protein-coding genes, 36 pseudogenes, 6 rRNAs (5S, 23S, and 16S), 50 aminoacyl-tRNA synthases, and 4 noncoding RNAs (ncRNAs). Functional classifications of these predicted genes were performed using the Integrated Microbial Genomes system (13).

In particular, the MAI of ME-1 was located at nucleotide (nt) position 422080 to 519743 in the genome. The MAI was 97,664 bp in length with a G+C content of 61.18%, lower than that of the genome. In total, 96 genes were predicted in the MAI region of ME-1 and 37 magnetosome-specific genes were clustered into five operons, including *mamCDFG*, *mms*, *mamAB*, *mamXY*, and an *mamAB*-like operon. The gene sequences of the *mamAB*-like operon (*mamEJOPAQRB*) are highly similar to those in the *mamAB* operon. In addition, an *mamW* and another *mamF* were deviated from these operons.

A cobalt-zinc-cadmium resistance system (*WV31_01800-01815*) and a ferrous iron transport system (*feo*) (*WV31_01745-01750*) were also found in the MAI. Additionally, another *feo* system and three ferric uptake regulator-like genes were identified in the genome. These genes may play roles in the biomineralization of magnetosomes.

Further investigations into the genome of ME-1 may enhance the understanding of the formation of magnetosomes and facilitate the applications of MTB in a broad range of fields.

### Accession number(s).

The genome sequence of strain ME-1 has been deposited in NCBI GenBank under the accession number CP015848.
